# Intrapancreatic fat deposition shows distinct regional and compartmental associations with obesity and diabetes

**DOI:** 10.1016/j.isci.2026.117292

**Published:** 2026-08-24

**Authors:** Darbaz Adnan, Abdullah Allaoa, Edena R. Khoshaba, Lin Cheng, Faraz Bishehsari

**Affiliations:** 1 Rush Center for Integrated Microbiome and Chronobiology Research, Rush Medical College, Rush University Medical Center, Chicago, IL, USA; 2 Gastroenterology Research Center (GRC), Department of Internal Medicine, University of Texas Health Science Center at Houston, Houston, TX, USA; 3 MD Anderson Cancer Center, UTHealth Houston Graduate School of Biomedical Sciences, Houston, TX, USA

**Keywords:** intrapancreatic fat deposition, obesity, diabetes, fatty pancreas disorder

## Abstract

The objective of this study is to identify determinants of fat deposition in anatomically distinct regions of the pancreas. One hundred donor pancreases without active pancreatic cancer were histologically examined across distinct regions for intralobular and interlobular fat. Predictors of fat deposition in the pancreatic head and tail were determined. Tail intralobular fat was greater than head intralobular fat, and female donors had greater fat across all measured regions and compartments. The distribution of fat in the head and tail varied depending on the presence of obesity or diabetes. Patterns of intrapancreatic fat deposition (IPFD) and associated risk factors can vary by anatomical region.

## Highlights

• Intrapancreatic fat deposition varied across regions and compartments
• Female sex was associated with greater fat across all measured compartments
• Obesity was most strongly associated with head interlobular fat
• Type 2 diabetes was associated with tail intralobular fat

## Graphical abstract

**Figure:**
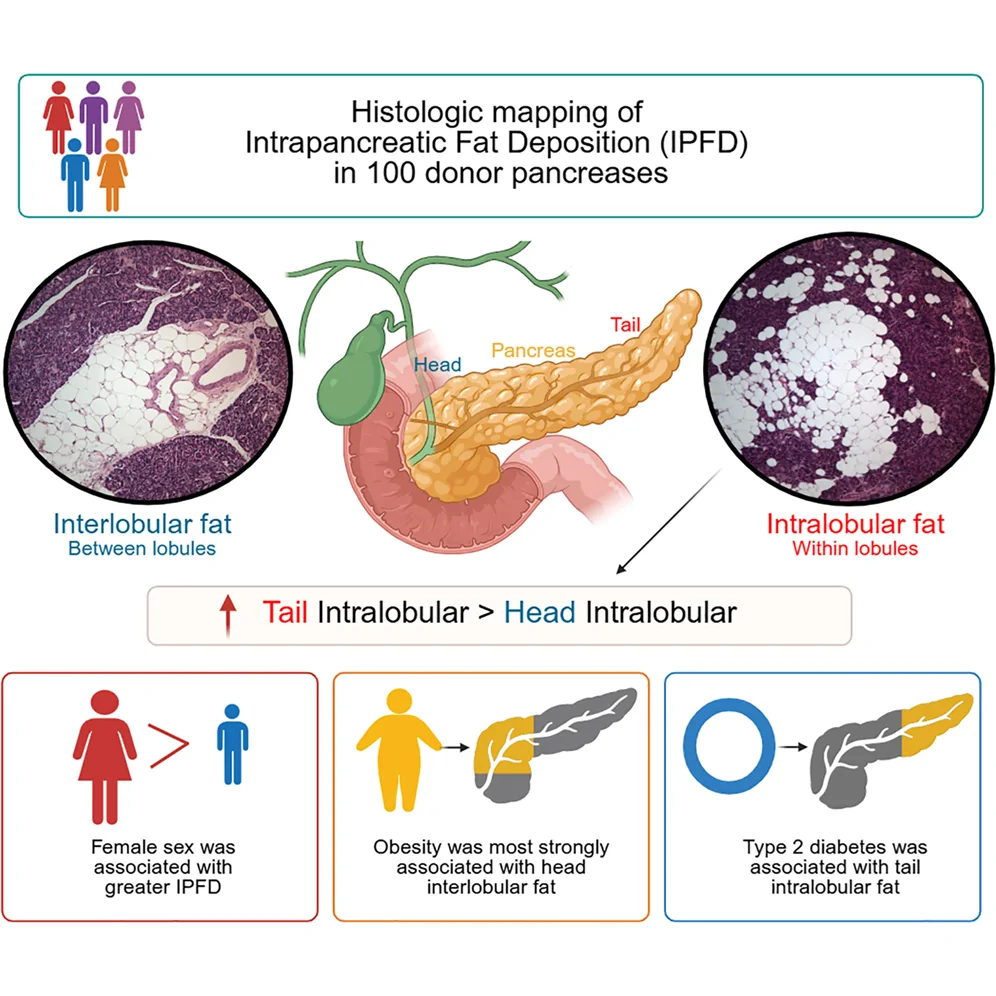

## Teaser

biological sciences; biochemistry; diabetology

## Introduction

Intrapancreatic fat deposition (IPFD), characterized by fat deposition within the pancreas, is commonly detected on imaging and is clinically significant.^1^ When diffuse and excessive, this fat deposition is increasingly recognized as fatty pancreas disorder (FPD) that has functional, pathophysiological, and prognostic significance.^2^^,^^3^ IPFD is associated with an increased risk of pancreatic diseases, including diabetes, pancreatitis, and cancer.^4^^,^^5^^,^^6^^,^^7^^,^^8^^,^^9^ These emerging associations underscore the need for better characterization of IPFD and its causes and clinical implications.

Recent multidisciplinary consensus recommends fatty pancreas as the umbrella clinical term for pancreatic fat deposition; in the present study, however, we use IPFD to denote the histologically quantified adipocyte-area outcome.^10^ Pancreatic fat accumulates in various compartments, including interlobular (between lobules), intralobular (within lobules), and intracellular fat, reflecting remodeling processes best assessed histologically.^11^^,^^12^^,^^13^ Pancreatic fat depots exhibit biologically important anatomical variations.^14^ However, factors that influence preferential fat deposition in specific compartments or anatomical regions, such as the head versus the tail, remain poorly understood. To study fat distribution across anatomically distinct regions, we prospectively sampled the proximal (head) and distal (tail) ends of the pancreas from deceased organ donors, selecting these regions *a priori* to ensure clear anatomical separation and reproducible sampling. Tissue blocks were obtained from these two ends prior to formalin fixation, paraffin embedding (FFPE), and sectioning.

Here, we performed blinded histologic quantification of interlobular and intralobular fat in anatomically distinct head and tail regions from 100 donor pancreases without active pancreatic cancer. By integrating site-specific fat measurements with donor demographic and clinical variables, we found that pancreatic fat distribution is compartment- and region-dependent, with sex emerging as a consistent correlate, body mass index (BMI) showing a stronger association with head interlobular fat, and diabetes showing a stronger association with tail intralobular fat. These data refine current understanding of pancreatic fat heterogeneity by linking clinical correlates to anatomically distinct histologic compartments in a large donor-based cohort.

## Results

### Pancreatic fat differs across compartments and regions and is higher in female donors

**Figure 1:**
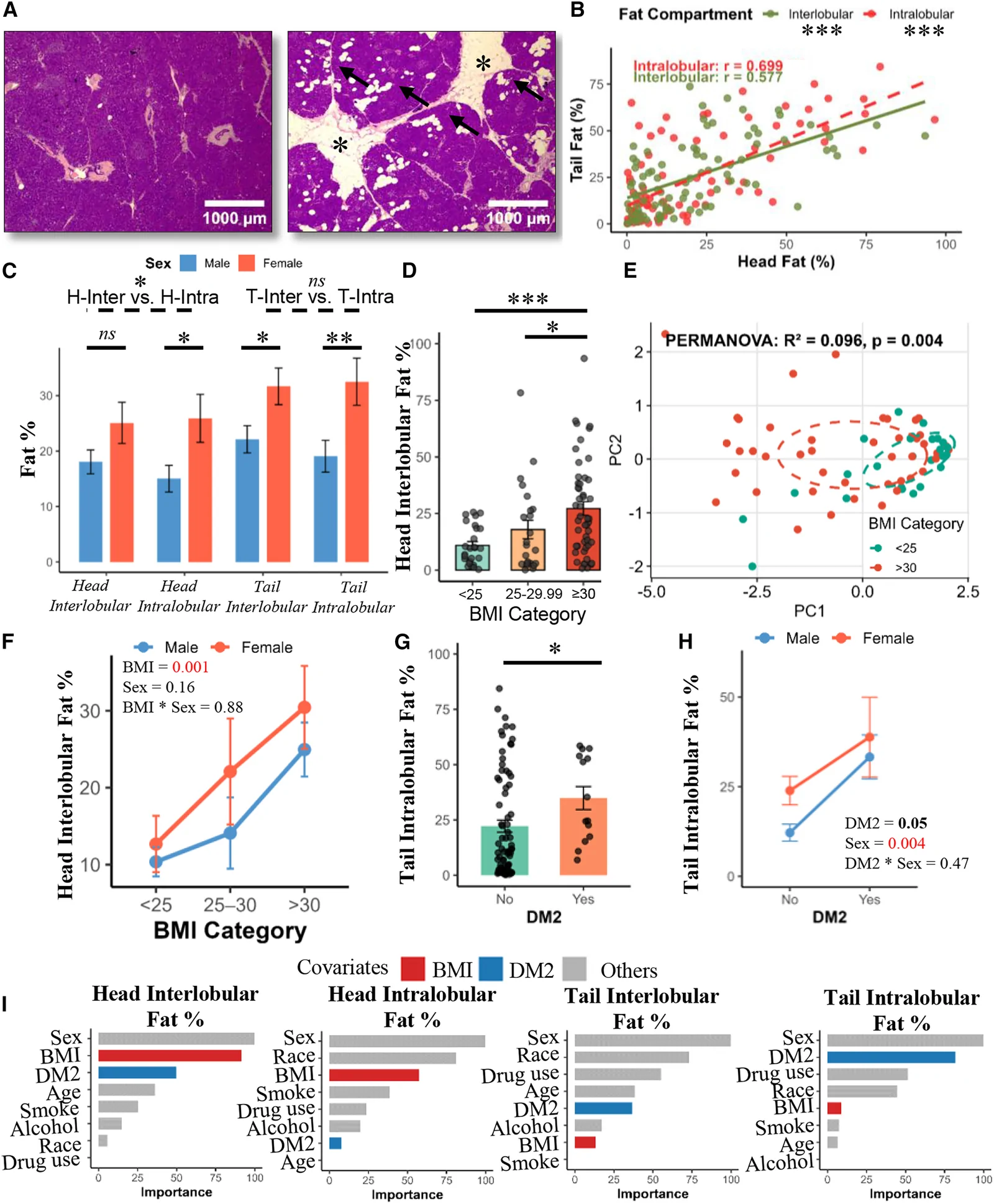
Regional and compartment-specific IPFD differ by sex and show distinct associations with obesity and diabetes (A) Representative H&E-stained pancreatic sections showing variation in fat deposition. The left image depicts a pancreas with minimal to no fat infiltration, while the right image shows extensive IPFD. Asterisks (∗) indicate interlobular fat deposition in connective tissue between lobules, while arrows highlight intralobular fat deposits located within the lobular parenchyma. Scale bars, 1,000 μm. (B) Correlation between head and tail fat for each compartment. Both tail intralobular and interlobular fat was statistically significantly correlated with head intralobular and interlobular fat (r = 0.699, *p* < 0.001, r = 0.577, *p* < 0.001, respectively). (C) Fat percentage across pancreatic regions and compartments stratified by sex. Sex differences within each region-compartment group were assessed using pairwise estimated marginal mean contrasts from a linear mixed-effects model with donor as a random effect and region, compartment, and sex as fixed effects, with Holm adjustment for multiple comparisons. Head-versus-tail comparisons were assessed separately within donors after Shapiro-Wilk testing of paired differences, using a paired *t* test for interlobular fat and a Wilcoxon signed-rank test for intralobular fat. Female donors had significantly higher fat deposition in all regions and compartments compared with male donors. Bars represent mean ± SEM. Tail intralobular fat was significantly greater than head intralobular fat. H-Inter, head interlobular; H-Intra, head intralobular; T-Inter, tail interlobular; T-Intra, tail intralobular. (D) Head interlobular fat across BMI categories. Overall differences across BMI categories were assessed using the Kruskal-Wallis test, followed by Dunn’s post hoc test with Benjamini-Hochberg adjustment for pairwise comparisons. Individuals with BMI ≥ 30 kg/m^2^ had significantly higher fat deposition compared with those with lower BMI (25–29.99 and <25 kg/m^2^). Bars represent mean ± SEM and individual data points. (E) Principal component analysis (PCA) of pancreatic fat composition based on site and compartments (head and tail interlobular and intralobular), colored by BMI category (<25 and ≥30 kg/m^2^). PERMANOVA test indicates that BMI significantly contributes to the variation in fat deposition patterns (R^2^ = 0.096, *p* = 0.004). (F) Head interlobular fat increases across BMI categories in both sexes. two-way ANOVA *p* values are shown for BMI, sex, and their interaction. (G) Tail intralobular fat was statistically significantly higher in individuals with type 2 diabetes mellitus (DM2) compared with those without. Bars represent mean ± SEM; individual values are overlaid. (H) Sex-specific analysis showing that tail intralobular fat was higher in sexes with DM2 compared with individuals with no history of DM2. Two-way ANOVA *p* values are shown for DM2, sex, and their interaction. (I) Variable importance analysis from linear regression models predicting fat deposition by compartments. Sex consistently ranked as the most important predictor across all compartments. BMI (red) had higher importance than DM2 (blue) in the pancreatic head for both interlobular and intralobular fat, with BMI ranking second for head interlobular fat. In contrast, DM2 was more important than BMI in the pancreatic tail, ranking second for tail intralobular fat. In all figures, fat % represents the mean adipocyte area fraction within the sampled histologic region of interest (ROI) for the indicated compartment and anatomic region, rather than the percentage of fat in the whole pancreas. For all images, asterisks denote significance (∗*p* < 0.05, ∗∗*p* < 0.01, ∗∗∗*p* < 0.001, and ns denotes not significant).

We analyzed pancreas samples from 100 adult donors (62% male; mean age, 45.6 years; mean BMI, 30.7 kg/m^2^ and 38% female; mean age, 49.4, BMI, 32 kg/m^2^). Demographics and clinical factors were comparable between sexes (Table 1). Overall, 51 donors met criteria for obesity (BMI ≥ 30 kg/m^2^). Fat was measured in the interlobular and intralobular compartments (Figure 1A), with the assessment blinded to donor identity. Fat fraction was quantified in ImageJ as adipocyte area divided by total tissue area in systematically sampled hematoxylin and eosin (H&E) fields. We use IPFD to denote overall pancreatic fat deposition, whereas interlobular fat refers to adipose tissue located in septal/connective tissue between lobules, and intralobular fat refers to adipocytes embedded within lobular parenchyma.^15^ Cause of death was abstracted from the donor research chart and is reported in Table 1.

**Table 1:** Demographic data separated by sex

Variable	Male	Female	Overall	Univariate statistical analysis
	(*n* = 62)	(*n* = 38)	(*n* = 100)	
Age (years)				
Mean (SD)	45.6 (13.9)	49.4 (13.4)	47.0 (13.8)	Welch two sample *t*
Median [Min, Max]	46.0 [18.0, 76.0]	51.0 [18.0, 74.0]	48.0 [18.0, 76.0]	Test. *t* = −1.38, *p* = 0.168
BMI (kg/m^2^)				
Mean (SD)	30.7 (9.60)	32.0 (8.01)	31.2 (9.02)	Wilcoxon rank-sum test
Median [Min, Max]	29.4 [15.7, 58.9]	30.9 [18.7, 57.7]	30.1 [15.7, 58.9]	W = 1029, *p* = 0.291
Race				
White	23 (37.1%)	20 (52.6%)	43 (43.0%)	Fisher’s exact test
Hispanic/Latino	11 (17.7%)	6 (15.8%)	17 (17.0%)	*p* = 0.519
Black	23 (37.1%)	9 (23.7%)	32 (32.0%)	
Asian	3 (4.8%)	3 (7.9%)	6 (6.0%)	
American Indian	1 (1.6%)	0 (0%)	1 (1.0%)	
Other	1 (1.6%)	0 (0%)	1 (1.0%)	
Smoking				
Yes	31 (50.0%)	16 (42.1%)	47 (47.0%)	Pearson’s chi-squared
No	31 (50.0%)	22 (57.9%)	53 (53.0%)	χ^2^ = 0.315, *p* = 0.574
DM2				
Yes	12 (19.4%)	5 (13.2%)	17 (17.0%)	Pearson’s chi-squared
No	50 (80.6%)	33 (86.8%)	83 (83.0%)	χ^2^ = 0.277, *p* = 0.598
Illicit drug use				
Yes	21 (33.9%)	9 (23.7%)	30 (30.0%)	Pearson’s chi-squared
No	41 (66.1%)	29 (76.3%)	70 (70.0%)	χ^2^ = 0.729, *p* = 0.393
Excessive alcohol use				
Yes	14 (22.6%)	6 (15.8%)	20 (20.0%)	Pearson’s Chi-squared
No	48 (77.4%)	32 (84.2%)	80 (80.0%)	χ^2^ = 0.32, *p* = 0.571
Cause of death				
Anoxia	26 (41.9%)	14 (36.8%)	40 (40.0%)	Fisher’s Exact Test
CVA^a^/stroke	21 (33.9%)	13 (34.2%)	34 (34.0%)	*p* = 0.783
Trauma/head injury	14 (22.6%)	9 (23.7%)	23 (23.0%)	
Other/unknown^a^	1 (1.6%)	2 (5.3%)	3 (3.0%)	

BMI, body mass index; CVA, cerebrovascular accident; DM2, type 2 diabetes mellitus. Trauma/head injury includes head trauma and trauma/blunt injury. Other/unknown includes CNS tumor and missing cause-of-death data.

a Values are presented as mean (SD), median [Min, Max], or *n* (%), as appropriate. Percentages are calculated within each sex-specific or overall column. Univariate comparisons between male and female donors were performed using Welch’s two-sample *t* test, Wilcoxon rank-sum test, Pearson’s chi-squared test, or Fisher’s exact test, as indicated.

Pancreatic head fat overall correlated with that in the tail across both intralobular and interlobular compartments (Pearson r = 0.69 and 0.57, *p* < 0.001; Figure 1B). While fatty changes in the tail were more pronounced than in the head, this difference reached statistical significance only in the intralobular compartment (*p* = 0.02, Figure 1C). However, intralobular and interlobular fat were not evenly distributed; some individuals showed predominantly intralobular fat deposition with minimal interlobular fat, and vice versa, highlighting the compartmental variability of fat deposition even within the same individuals. Overall, female donors exhibited significantly greater IPFD than male donors across regions and compartments (Figure 1C). Descriptive results of interlobular, intralobular, and total pancreatic fat in the overall cohort and stratified by race are provided in Table S1.

### Obesity preferentially associates with head interlobular fat, whereas diabetes associates with tail intralobular fat

Among the four histologic fat measurements: head interlobular, head intralobular, tail interlobular, and tail intralobular (mean ± standard deviation [SD]) fat fractions were 20.70% ± 19.35%, 19.13% ± 22.49%, 25.77% ± 18.96%, and 24.19% ± 23.43%, respectively; total fat fraction was 19.95% ± 19.58% in the head and 24.97% ± 20.25% in the tail (Table S1). Metabolic conditions like obesity and type 2 diabetes mellitus (DM2) contribute to pancreatic fat deposition. In our analysis, donors with obesity (BMI ≥ 30 kg/m^2^) had the highest interlobular fat (Figure 1D). Intralobular fat did not differ statistically significantly across BMI categories. To summarize the four donor-level fat measurements jointly, we performed principal components analysis (PCA) as an exploratory multivariate analysis, which showed significant clustering by BMI category on permutational multivariate analysis of variance (PERMANOVA) testing (PERMANOVA R^2^ = 0.096, *p* = 0.004; Figure 1E). In a two-way ANOVA with sex and BMI (Figure 1F), BMI independently predicted head interlobular fat (*p* = 0.001). No evidence of a BMI-by-sex interaction was observed in this cohort. BMI category was not significantly associated with tail fat measures in this cohort. On the other hand, donors with diabetes had significantly greater tail intralobular fat than donors without diabetes (34.8% vs. 22.19%, *p* = 0.015; Figure 1G), even after adjusting for sex (Figure 1H), whereas fat in the head (interlobular or intralobular) did not differ by diabetes status. Figure 1G shows the unadjusted comparison of tail intralobular fat by diabetes status, whereas Figure 1H presents the same outcome stratified by sex and analyzed using a two-way ANOVA. Interestingly, obesity was not significantly associated with tail fat measures, and diabetes status was not significantly associated with head interlobular fat (all *p* > 0.05). This compartment-specific link is consistent with prior observations from pancreas imaging that fat in the pancreatic tail correlated with insulin resistance.^7^ We observed no statistically significant correlation between HbA1c and intrapancreatic fat in any compartment (r = −0.06 to 0.28, all *p* ≥ 0.27; Figure S1), both in the overall cohort and in individuals with DM2 (*n* = 17). Additionally, in a subgroup analysis stratified by obesity status, we observed no significant associations between intra- or interlobular fat and HbA1c levels, or with the use of glucose- or lipid-lowering medications (all *p* > 0.05; Table S2).

### Multivariable models identify sex as the dominant predictor and reveal region-specific contributions of BMI and diabetes

We next conducted regression analyses to identify independent predictors of fat deposition using three models. Model 1 adjusted for sex, race/ethnicity, BMI, and DM2. Model 2 added age to these covariates, and Model 3 further included behavioral factors, history of alcohol use, recreational drug use, and tobacco smoking for head (Table 2) and tail (Table 3) regions. Sex consistently emerged as the most dominant predictor of fat content across all models. BMI was significantly associated with head interlobular fat in all models, while DM2 showed an association with tail intralobular fat across models. Notably, White and Hispanic donors had higher pancreatic fat than Black donors.

**Table 2:** Multiple regression analysis for head of pancreas regions and compartment

Variable	Head Inter-lobular	Head Inter-lobular	Head Inter-lobular	Head Intra-lobular	Head Intra-lobular	Head Intra-lobular
	β-coefficient^a^	*p*	Importance	β-coefficient	*p*	Importance
Model 1^a^						
(Intercept)	−3.024	0.7082	–	−4.531	0.6282	–
Sex (female)	10.514	**0.0134**	**100**	12.304	**0.0125**	100
BMI (kg/m^2^)	0.604	**0.0206**	**92.95**	0.411	0.1687	42.97
DM2 (yes)	6.43	0.2429	42.63	3.229	0.6113	0
Race: Hispanic	2.635	0.6506	11.97	5.598	0.4066	15.85
Race: White	−0.773	0.8632	0	10.889	**0.0384**	77.96
Model 2^a^						
(Intercept)	3.473	0.726	–	−3.878	0.737	–
Sex (female)	11.166	**0.0093**	**100**	12.369	**0.0134**	100
BMI (kg/m^2^)	0.624	**0.0169**	**91.16**	0.413	0.1702	52.9
DM2 (yes)	7.859	0.1643	50.5	3.372	0.6071	17.22
Race: Hispanic	3.025	0.6032	15.85	5.637	0.4069	30.28
Race: White	−0.531	0.9057	0	10.913	**0.0394**	82.1
Age (years)	−0.166	0.2596	39.96	−0.017	0.9224	0
Model 3^a^						
(Intercept)	0.534	0.9634	–	−13.337	0.3185	–
Sex (female)	11.448	**0.0088**	**100**	12.971	**0.0095**	100
BMI (kg/m^2^)	0.637	**0.0184**	**89.17**	0.477	0.1199	56.01
DM2 (yes)	7.315	0.2057	45.64	2.258	0.732	6.2
Race: Hispanic	4.443	0.4693	24.56	9.341	0.1862	46.35
Race: White	−0.425	0.9279	0	11.997	**0.0281**	83
Age (years)	−0.152	0.322	34.88	0.033	0.8493	0
Smoke (Yes)	3.967	0.4187	27.811	6.757	0.2306	41.25
Drug use (Yes)	0.631	0.9038	1.17	4.984	0.4058	26.16
Excessive alcohol use (Yes)	−2.827	0.5869	17.51	−4.517	0.4492	23.11

BMI, body mass index; DM2, type 2 diabetes mellitus.

β coefficients represent the estimated change in fat percentage associated with each predictor, relative to the indicated reference group for categorical variables. Variable-importance scores were derived from the corresponding linear models and are presented as scaled relative importance values. coefficients represent the estimated change in fat percentage associated with each predictor, relative to the indicated reference group for categorical variables. Variable-importance scores were derived from the corresponding linear models and are presented as scaled relative importance values. Bold entries indicate covariates with p < 0.05 and their corresponding importance scores.

a Three ordinary least-squares linear regression models were used to evaluate predictors of head interlobular and head intralobular fat percentage. Model 1 included sex, BMI, DM2, and race. Model 2 additionally included age. Model 3 additionally included smoking, illicit drug use, and excessive alcohol use.

**Table 3:** Multiple regression analysis for tail of pancreas regions and compartment

Variable	Tail Inter-lobular	Tail Inter-lobular	Tail Inter-lobular	Tail Intra-lobular	Tail Intra-lobular	Tail Intra-lobular
	β coefficient^a^	*p*	Importance	β coefficient	*p*	Importance
Model 1^a^						
(Intercept)	12.349	0.1319	–	8.154	0.401	–
Sex (Female)	10.943	**0.0143**	100	16.55	**0.0021**	100
BMI (kg/m^2^)	0.095	0.7151	0	0.081	0.7926	0
DM2 (yes)	7.972	0.1874	45.02	18.904	**0.0098**	81.66
Race: Hispanic	11.105	0.0692	68.98	9.433	0.1925	35.99
Race: White	7.796	0.0978	61.18	7.381	0.1866	36.6
Model 2^a^						
(Intercept)	7.106	0.485	–	6.53	0.591	–
Sex (female)	10.544	**0.019**	100	16.426	**0.0026**	100
BMI (kg/m^2^)	0.083	0.748	0	0.078	0.8027	0.9
DM2 (yes)	6.92	0.2621	38.89	18.578	**0.0133**	79.72
Race: Hispanic	10.819	0.0775	70.69	9.345	0.2002	36.84
Race: White	7.567	0.1091	62.58	7.31	0.1944	37.42
Age (years)	0.129	0.3887	26.23	0.04	0.823	0
Model 3^a^						
(Intercept)	−4.325	0.717	–	−6.337	0.6566	–
Sex (female)	10.833	**0.0155**	100	16.768	**0.002**	100
BMI (kg/m^2^)	0.195	0.462	17.05	0.202	0.523	11.33
DM2 (yes)	7.273	0.243	37.91	19.388	**0.0105**	79.8
Race: Hispanic	13.271	**0.0371**	83.07	12.642	0.0945	47.62
Race: White	9.799	**0.0433**	79.88	10.039	0.0818	50.06
Age (years)	0.199	0.1967	43.91	0.118	0.5181	11.594
Smoke (yes)	−1.972	0.7036	0	−3.192	0.6063	7.05
Drug use (yes)	9.639	0.082	65.87	12.944	0.0512	57.58
Excessive alcohol use (yes)	3.632	0.4929	14.64	1.97	0.7551	0

BMI, body mass index; DM2, type 2 diabetes mellitus. Bold entries indicate covariates with p < 0.05 and their corresponding importance scores.

a Three ordinary least-squares linear regression models were used to evaluate predictors of tail interlobular and tail intralobular fat percentage. Model 1 included sex, BMI, DM2, and race. Model 2 additionally included age. Model 3 additionally included smoking, illicit drug use, and excessive alcohol use. β coefficients represent the estimated change in fat percentage associated with each predictor, relative to the indicated reference group for categorical variables. Variable-importance scores were derived from the corresponding linear models and are presented as scaled relative importance values.

Variable importance analysis confirmed the association between several previously suggested risk factors and IPFD, although the strength of these associations varied across different regions and compartments. Sex remained the strongest covariate of fatty deposition across pancreases (Tables 2 and 3). Obesity carried greater weight than diabetes in predicting fat deposition in the pancreatic head, whereas diabetes showed a higher weight than obesity in contributing to fat deposition in the tail (Figure 1I; Tables 2 and 3).

## Discussion

In this study, we demonstrate that intrapancreatic fat deposition is region- and compartment-specific rather than uniformly distributed throughout the gland. Using histologic quantification across anatomically distinct pancreatic regions in a large donor cohort, we show that clinical and metabolic correlates of pancreatic fat vary according to both location and compartment. These findings provide further evidence that pancreatic fat deposition represents a heterogeneous process that may reflect distinct biological mechanisms across different regions of the pancreas.

Building upon prior IPFD studies, our work examines a large contemporary donor cohort using blinded, compartment-specific quantification in anatomically prespecified head and tail regions and links these measurements to clinical and metabolic correlates. Our findings extend prior surgical pancreatectomy series,^16^ in which the majority of specimens were obtained from pancreatic ductal adenocarcinoma cases and demonstrated heterogeneous intrapancreatic fat distribution, even when sampling within a single pancreatic region. By incorporating site- and compartment-specific assessments of non-resected donor pancreata from a cancer-free population, our study provides a broader representation of intrapancreatic fat distribution in the noncancer donors and its relationship to clinical factors, including obesity and diabetes. Our findings support the concept that metabolic dysfunctions such as obesity and diabetes contribute to compartment-specific IPFD. However, their impact may vary depending on the specific region of the pancreas.^7^ Interlobular fat, particularly in the pancreatic head, appeared to reflect systemic fat deposition within the interlobular septa. Likewise, age showed a greater relative contribution to the interlobular than to the intralobular fat in the variable-importance analysis (Figure 1I). These findings highlight the significance of region-specific fat distributions in the pancreas. Recent imaging work suggests that pancreatic fat in the head is associated with BMI, visceral fat, and metabolic-syndrome parameters, whereas fat in the tail has shown closer associations with insulin resistance and diabetes-related traits.^14^ Because the tail also contains a relatively higher endocrine/islet density (>2-fold higher) than the head,^17^ IPFD in this region may more closely track diabetes-associated parenchymal remodeling. These interpretations remain hypothesis-generating and require direct mechanistic validation.

Our observation of greater pancreatic fat in female donors should be interpreted in the context of prior mixed literature, as some studies have reported higher pancreatic fat in men, while others suggest stronger metabolic correlations in women. In our cohort, female donors were also modestly older than male donors on average, which may have contributed to the observed difference.^14^^,^^18^ Our findings are nevertheless consistent with prior studies suggesting that fat distribution across metabolic organs, including the liver and pancreas, may be sex-specific.^19^^,^^20^ In particular, women have been reported to exhibit greater pancreatic fat deposition in certain settings,^19^^,^^20^ although other studies have reported the opposite pattern.^20^ Emerging evidence further suggests that sex-related differences in pancreatic fat deposition may be context dependent, with stronger associations between IPFD and obesity, visceral adiposity, and subcutaneous adiposity observed in women in some cohorts.^14^

The observed racial/ethnic differences in pancreatic fat should be interpreted cautiously. Our dataset lacked the detailed body-composition and socio-environmental measures needed to evaluate mechanism, but prior studies have suggested that adipose partitioning differs across racial/ethnic groups, including lower visceral adiposity at a given BMI in some Black cohorts compared with White or Hispanic cohorts. Accordingly, our findings should be viewed as preliminary and as motivation for more deeply phenotyped future studies.^21^ Prior histologic studies^22^^,^^23^ have largely characterized pancreatic fat in older populations, where advanced age itself is a major determinant of fat deposition. In contrast, the relative enrichment of younger and middle-aged donors in our cohort provides complementary insight into regional pancreatic fat distribution earlier in adulthood, before the substantial age-associated pancreatic fat deposition described in prior autopsy and donor studies. A classic autopsy series demonstrated that pancreatic fat deposition was common and associated with age and overweight, whereas a recent study of 36 adult donor pancreases using dense 16-region sampling described distinct adipocytic and fibrotic phenotypes in DM2.^23^ Relative to these studies, our work adds a larger donor cohort with blinded, compartment-specific quantification in anatomically prespecified head and tail regions, demonstrating that clinical correlates of pancreatic fat are region- and compartment-specific rather than uniform across the gland.

### Limitations of the study

A potential limitation of our fat quantification is the heterogeneous (spotty) distribution of adipocytes within pancreatic tissue. We attempted to mitigate this by systematically sampling predefined fields across each section. In addition, our histologic approach focused on adipocyte infiltration (IPFD) as assessed on H&E staining and therefore does not capture intracellular lipid droplets or distinguish between physiological fat deposition and FPD.^19^ Accordingly, the underlying mechanism of pancreatic adipocyte accumulation cannot be determined from the present study.^10^^,^^24^

The de-identified donor research charts might have lacked information regarding prior pancreatic disease, such as pancreatitis, medication exposure, and longitudinal weight history, all of which may influence pancreatic fat deposition and therefore represent potential unmeasured confounders. In particular, medication data were not sufficiently granular to evaluate class-specific effects of glucose-lowering therapies on IPFD.^25^

Only 17 donors had DM2, and information regarding diabetes subtype, disease duration, and longitudinal glycemic history was unavailable. Consequently, diabetes-related associations should be interpreted cautiously. Likewise, the absence of significant correlations between HbA1c and regional fat measures should be interpreted in the context of HbA1c reflecting relatively recent glycemic exposure rather than long-term metabolic history.

Finally, although sampling was intentionally performed in anatomically distinct head and tail regions to maximize reproducibility, these measurements do not capture the entire pancreas. In addition, all histologic assessments were performed by a single-blinded observer, and inter-observer reproducibility was not formally evaluated. Future studies integrating whole-gland sampling, imaging-based fat quantification, and independent histologic validation will be important for further characterization of regional pancreatic fat distribution.

## Resource availability

### Lead contact

Requests for further information and resources should be directed to and will be fulfilled by the lead contact, Faraz Bishehsari (faraz.bishehsari@uth.tmc.edu).

### Materials availability

This study did not generate new unique reagents.

### Data and code availability

The de-identified donor-level data supporting the findings of this study are available from the lead contact upon reasonable request, subject to institutional review and any restrictions associated with sharing deceased-donor data. This paper does not report on new custom software. The R scripts used for statistical analyses and figure generation are available from the lead contact upon reasonable request. Any additional information required to reanalyze the data reported in this paper is available from the lead contact upon request.

## Acknowledgments

F.B. would like to acknowledge NIH support, CA27948, CA277110, and AG08614. The authors would like to sincerely thank the Organ Allocation Team at Gift of Hope (GOH) for their invaluable support and coordination in facilitating the allocation of normal pancreas tissue for this research project. The authors are especially grateful to Jerome Schillaci, Kristal Baldocchi, Jessina Macon, Cynthia Bertulis, Lisa DeLuca, Norberto Perez, Magda Umbao, Barbara Thomas, Grant Schumacher, and all other GOH organ allocation coordinators whose dedication and assistance were essential to the success of this study. The authors acknowledge the members of the Center for Integrated Microbiome and Chronobiology Research (CIMCR), particularly Ms. Denise Labedz, for their continued support to this project.

## Author contributions

D.A. dissected all pancreas samples, performed imaging and histological assessments, conducted data analysis and visualization, and drafted the manuscript. A.A. performed imaging and histological assessments, performed data analysis, and provided revisions to the manuscript. E.K. retrieved clinical data from electronic medical records of deceased donors. L.C. contributed to the histological assessment and interpretation of pancreatic samples. F.B. supervised the project, conceptualized and designed the study, guided study analysis, and critically revised the manuscript. All authors reviewed and approved the final version of the manuscript.

## Declaration of interests

The authors declare no competing interests.

## Declaration of generative AI and AI-assisted technologies in the writing process

During the preparation of this work, the authors used ChatGPT OpenAI to assist with R-code development and language editing. All code, analyses, and text were reviewed and validated by the authors, who take full responsibility for the content of the article.

## STAR★Methods

### Key resources table

**Table:** 

REAGENT or RESOURCE	SOURCE	IDENTIFIER
**Biological samples**		

Human donor pancreas tissue from deceased adult organ donors	Gift of Hope (GOH)	N/A

**Chemicals, peptides, and recombinant proteins**		

University of Wisconsin preservation solution	GOH	N/A
Hematoxylin and eosin staining reagents	Vector Laboratories	H-3502

**Software and algorithms**		

ImageJ	National Institutes of Health	RRID:SCR_003070
R	R Foundation for Statistical Computing	v4.5.0 (2025-04-11 ucrt); RRID:SCR_001905
Dplyr	CRAN	RRID:SCR_016708
ggplot2	CRAN	RRID:SCR_014601
Ggpubr	CRAN	RRID:SCR_021139
Ggrepel	CRAN	RRID:SCR_017393
Caret	CRAN	RRID:SCR_021138
vegan	CRAN	RRID:SCR_011950

### Experimental model and study participant details

#### Human donor pancreas specimens

Human pancreatic tissue was obtained from 100 deceased organ donors through a not-for-profit organ donation organization (Gift of Hope). Donor pancreases were procured only when the organ was not used for transplantation, either because the pancreas was deemed unsuitable for transplant or because the transplant recipient list had been exhausted. Whole-organ pancreas recovery was performed by transplant surgeons. The cohort included adult donors 18–76 years of age (mean age 47.0 years), including 62 male and 38 female donors. Chart-recorded sex was available in the de-identified donor records, whereas gender identity was not available. Race/ethnicity was analyzed as recorded in the donor chart.

Authorization for organ donation and research use was obtained by Gift of Hope through its standard organ-donation procedures before release of de-identified tissue and donor-level data for research. The study was approved by the Institutional Review Board (IRB) at Rush University Medical Center and was determined to be non-human-subjects research involving deceased donors. Donors with active pancreatic cancer were excluded. Histories of prior pancreatitis or other non-neoplastic pancreatic diseases were not systematically available in the de-identified donor research charts and therefore could not be adjudicated as exclusion criteria.

De-identified donor research charts supplied by Gift of Hope were used as the source for demographic and clinical variables. The data were obtained by the Gift of Hope team during the donor’s hospitalization prior to procurement, using patient medical records at the hospital and family interviews to collect past medical and social history. These charts summarized donor age, charted sex, body mass index (BMI), race/ethnicity, past medical history, selected laboratory values, medications, smoking, alcohol intake, and documented substance-use history. The cohort included donors of both sexes; sex was treated as a prespecified biological variable in descriptive analyses, interaction models, and multivariable regression. Race/ethnicity was analyzed as recorded in the donor chart and was not reassigned by study personnel.

Excessive alcohol use was abstracted from the past medical history section of the donor chart. Donors documented as “social” alcohol users were not classified as having excessive alcohol use. Otherwise, the chart was reviewed for evidence of excessive drinking using prespecified sex-specific thresholds: more than four drinks on any day or more than 14 drinks per week for men, and more than three drinks on any day or more than seven drinks per week for women. Smoking status was classified as positive if any cigarette use was documented, regardless of frequency. Drug use was defined as any documented use of recreational substances not prescribed by a physician, including but not limited to cocaine, heroin, marijuana, or amphetamines. Diabetes mellitus was classified using a combined chart-and-laboratory approach and was considered present if the donor had documented diabetes in the past medical history, use of glucose-lowering medication or insulin, or HbA1c ≥ 6.5%.^26^ Donors who did not meet these criteria remained in the non-diabetes group including donors with prediabetes (HbA1c 5.7%–6.4%), defined according to current guideline criteria.^26^

Fifteen of 17 donors with diabetes met both chart and HbA1c criteria; the remaining two met criteria for DM2 based on HbA1c ≥ 6.5% alone. No donor was considered to have DM2 without either laboratory or documented clinical evidence. Because acute inpatient laboratory values in organ donors may reflect terminal critical illness and ICU management rather than baseline metabolic status, HbA1c was the only laboratory-derived metabolic marker used for disease classification, as it reflects average glycemia over the preceding two to three months and is less sensitive to short-term fluctuation than fasting or tolerance-based glucose testing.^27^^,^^28^

### Method details

Whole pancreases were preserved in cold University of Wisconsin (UW) solution and delivered to the laboratory within 16 h of cross-clamp time. On receipt, each pancreas was dissected into anatomically distinct regions selected *a priori* for clear spatial separation: the tail (the most distal part of the pancreas) was separated from the remainder of the gland, and the head (most proximal part of the pancreas) was then further bisected to expose the main pancreatic duct. Samples from the pancreatic head and tail were collected in consistent locations between donors, and were fixed in formalin, embedded in paraffin, sectioned, stained with hematoxylin and eosin (H&E), and examined by light microscopy.

For histologic compartmentalization, interlobular fat was defined as adipose tissue located in interlobular septa or other connective tissue spaces between adjacent pancreatic lobules, including adipose tissue contiguous with the septal, perivascular, or periductal stromal compartment. Intralobular fat was defined as adipocytes located within lobular parenchyma, interspersed among acinar structures and not simply representing contiguous extension of septal adipose tissue. These definitions were applied prospectively to both head and tail sections. To standardize image acquisition across the entire tissue section, each paraffin section was divided into three equal segments along its longest axis. Within each segment, one field representing interlobular fat and one field representing intralobular fat was acquired according to the histologic criteria above. Thus, each compartmental summary for a given anatomical region was based on three fields sampled across the full section rather than from a single hotspot. This structured distinction between interlobular and intralobular compartments is consistent with published histologic descriptions that separate adipose tissue expanding septal/interlobular spaces from adipocytes embedded within lobular parenchyma.^29^^,^^30^

Image acquisition and quantitative fat analysis were performed by one trained observer who was fully blinded to donor clinical information, anatomical region, and histologic compartment at the time of both image capture and image analysis. Prior to study initiation, the observer underwent structured training and calibration with a gastrointestinal pathologist experienced in pancreatic histopathology to ensure consistent compartment identification and area-based quantification. All subsequent analyses were performed by the single observer using a prespecified and standardized protocol. All quantification was performed in ImageJ. For each image, fat content was defined as the tissue area occupied by adipocytes relative to the total tissue area in that field, using the same thresholding and area-selection criteria across all samples. The percent fat area for each field was calculated as: percent fat area = (adipocyte area/total tissue area) × 100.

For each compartment within each anatomical region, the mean of the three corresponding fields was used as the regional fat fraction. This yielded four primary fat measurements per donor: head interlobular fat, head intralobular fat, tail interlobular fat, and tail intralobular fat. Because the H&E-based workflow was designed to quantify mature adipocyte accumulation, intracellular lipid droplets were not assessed in this study.

### Quantification and statistical analysis

Unless otherwise stated, n refers to the number of donors. Continuous variables were summarized as mean ± standard deviation (SD) in the text and tables. Figures displaying summary bars used mean ± standard error of the mean (SEM), as specified in the figure legends. Comparisons between two groups were performed using two-sample t-tests when appropriate, and comparisons across more than two groups were performed using analysis of variance (ANOVA). Correlations between fat content in distinct pancreatic regions were assessed using Pearson’s correlation coefficient. Missing data were retained as missing (NA) in the source dataset, and no imputation procedures were performed. Randomization was not applicable because this was an observational donor-based study.

For figure-level inferential testing, head interlobular fat in Figure 1F was analyzed using a two-way ANOVA with sex and BMI category as fixed effects together with the sex × BMI interaction term. Tail intralobular fat in Figure 1G was first compared between donors with and without diabetes using a Wilcoxon rank-sum test. Figure 1H then evaluated tail intralobular fat using a two-way ANOVA with diabetes status and sex as fixed effects together with the diabetes × sex interaction term. These analyses were used as focused univariate or interaction-based tests for individual outcomes, whereas independent predictive effects across all measured covariates were addressed in separate multivariable linear regression models.

For Figure 1C, donor-level fat measurements were reshaped into long format and analyzed using a linear mixed-effects model to account for repeated within-donor measurements [Fat ∼ Region ∗ Compartment ∗ Sex + (1 | Subject_ID)]. Sex differences within each region-compartment cell were evaluated using estimated marginal mean contrasts (emmeans) with Holm adjustment for multiple comparisons. In addition, paired regional comparisons between head and tail were assessed within donors after Shapiro-Wilk testing of paired differences. Interlobular fat was compared using a paired *t* test, whereas intralobular fat was compared using a Wilcoxon signed-rank test.

For Figure 1D, head interlobular fat across BMI categories (<25, 25–29.99, and ≥30 kg/m^2^) was compared using the Kruskal-Wallis test, followed by Dunn’s post hoc test with Benjamini-Hochberg adjustment for pairwise comparisons.

To examine multivariate structure in pancreatic fat deposition, principal component analysis (PCA) was performed on the four donor-level fat variables: head interlobular fat, head intralobular fat, tail interlobular fat, and tail intralobular fat. Only complete cases across these four variables were included in the PCA. Variables were centered and scaled (prcomp(…, scale. = TRUE)). For the reported PCA/PERMANOVA comparison shown in Figure 1E, donors in the BMI categories <25 and ≥30 kg/m^2^ were retained, whereas the intermediate 25–30 kg/m^2^ group was not included in the inferential PERMANOVA model. PERMANOVA was performed with veganadonis2 using Euclidean distance and BMI category as the sole fixed factor (fat_matrix ∼ BMI_Category). No random factors, blocking variables, or restricted permutation strata were included because each donor contributed a single multivariate observation composed of four summarized fat outcomes. The PCA figure was generated with ggplot2, with ggpubr and ggrepel used for presentation features; the ellipse overlay was included for visualization only and was not itself inferential. The adonis2 framework implements permutation-based testing on a dissimilarity matrix, and with Euclidean distances its tests are equivalent to an RDA/ANOVA framework; the function’s default permutation count is 999 unless otherwise specified.

Independent predictors of fat deposition were evaluated using separate ordinary least squares linear regression models for each of the four fat outcomes: head interlobular, head intralobular, tail interlobular, and tail intralobular fat percentages. Model 1 included sex, race/ethnicity, BMI, and diabetes mellitus. Model 2 added age to Model 1. Model 3 further added smoking status, drug use, and excessive alcohol use. This strategy was used to assess the stability of associations across increasing levels of covariate adjustment. In parallel, variable-importance analysis was performed with the caret package using linear regression models fit with train(), including sex, race, age, BMI, smoking, diabetes mellitus, excessive alcohol use, and drug use. Variable-importance analysis was used as a descriptive ranking approach to compare predictor contribution across compartments; for linear models in the caret framework, this metric is derived from rescaled absolute model statistics and should not be interpreted as a causal effect size. Final visualization was generated in ggplot2. For figure panels annotated with significance symbols, asterisks denote ∗*p* < 0.05, ∗∗*p* < 0.01, and ∗∗∗*p* < 0.001, and ns denotes not significant. All tests were two-sided, and *p* < 0.05 was considered statistically significant. Statistical analyses were performed in R version 4.5.0 (2025-04-11 ucrt).
