# Supplementary material for: Intrapancreatic fat deposition shows distinct regional and compartmental associations with obesity and diabetes
Source: iScience. 2026 Aug 24;29(9):117292. doi: 10.1016/j.isci.2026.117292 (PMC13524749; doi:10.1016/j.isci.2026.117292)
Supplement: Supplementary file 1 [file mmc1.pdf]

iScience, Volume *29*

Supplemental information

Intrapancreatic fat deposition shows distinct regional and compartmental associations with obesity and diabetes

Darbaz Adnan, Abdullah Allaoa, Edena R. Khoshaba, Lin Cheng, and Faraz Bishehsari

Figure S1: IPFD and HbA1C in donors and a subset of donors with diabetes.

A) HbA1C and IPFD in all population (n = 100)

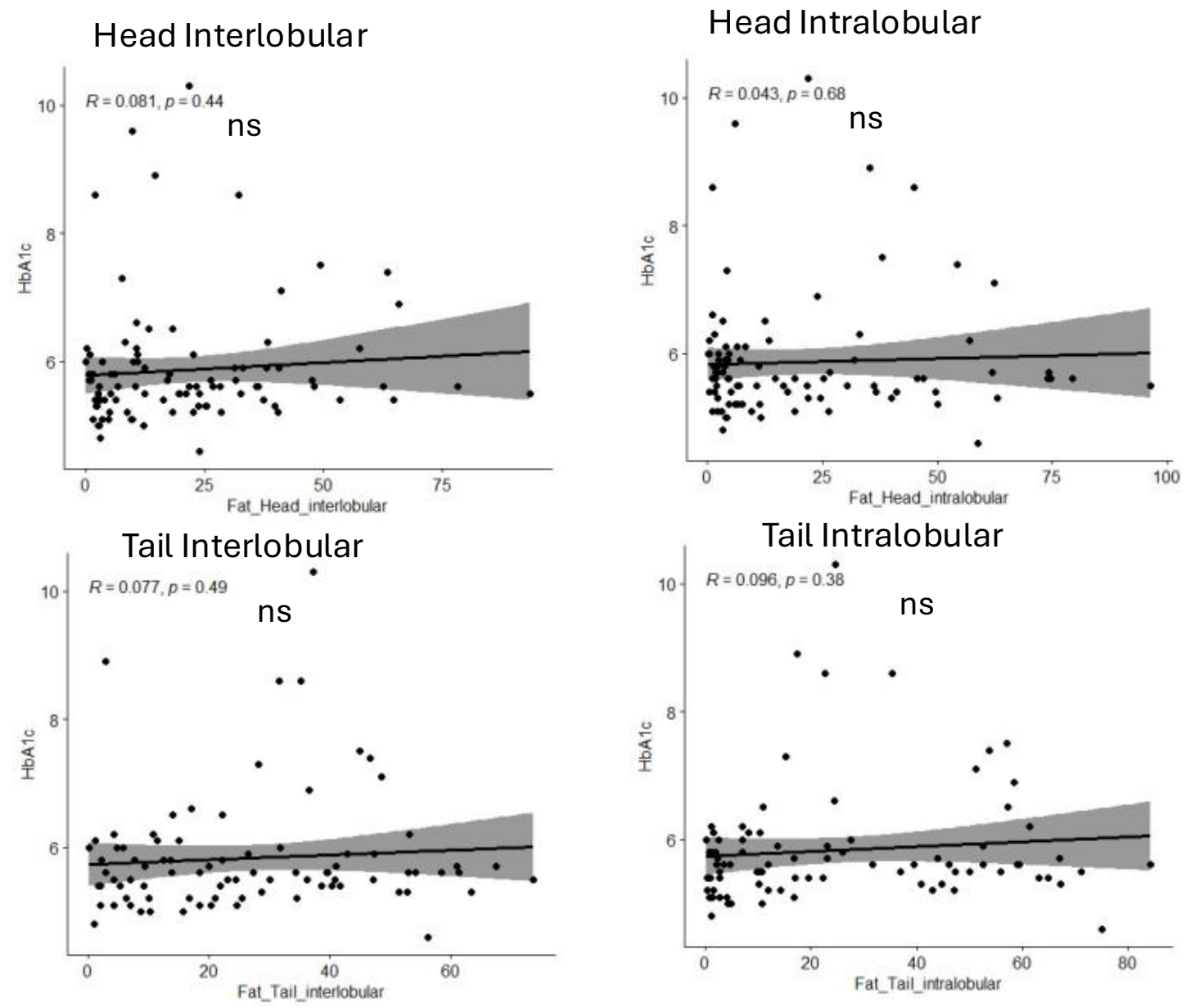

B) HbA1C and IPFD in diabetic subjects only(n = 17)

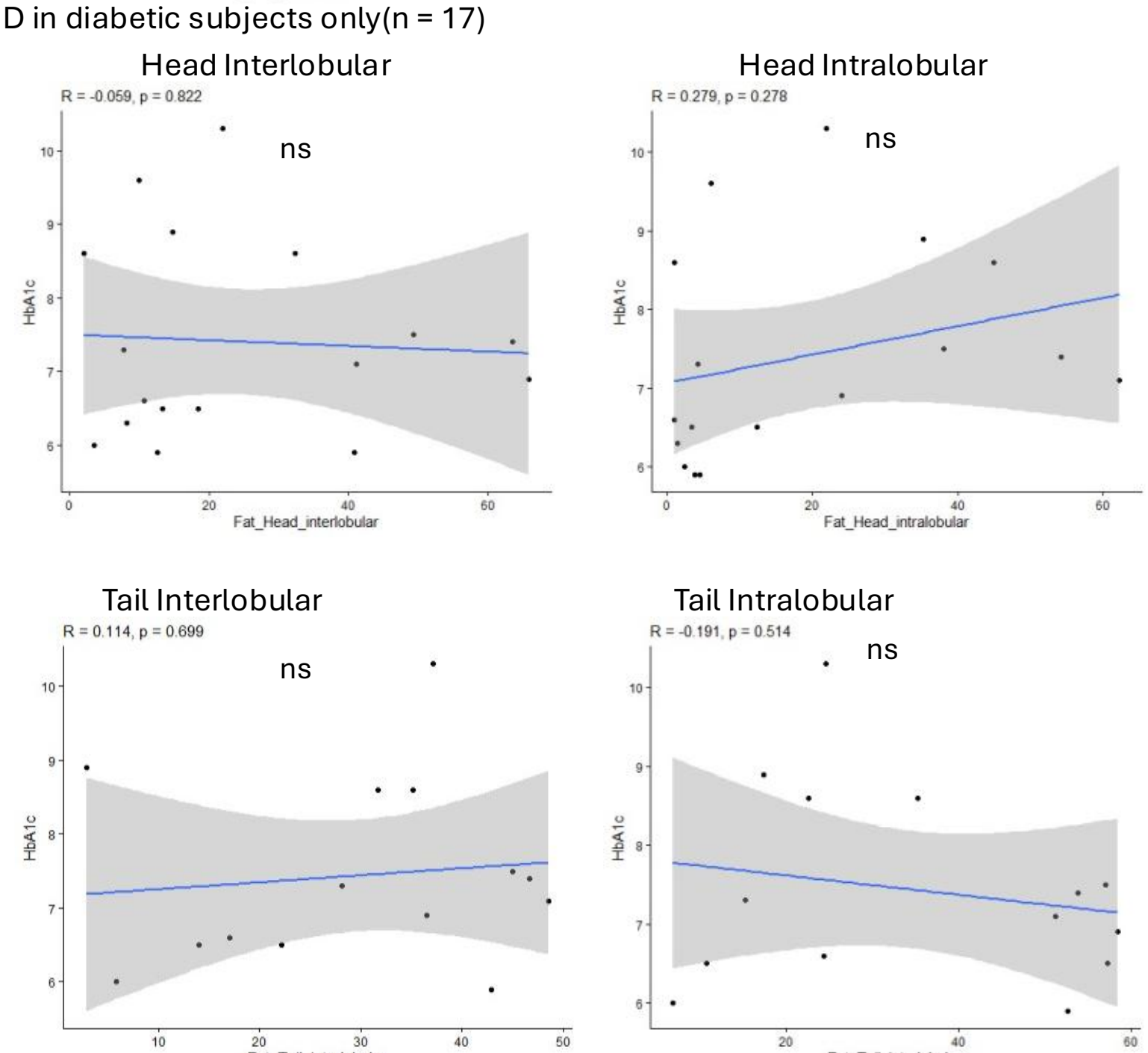

Table S1: IPFD stratified by Race

Race	Head interlobular	Head intralobular	Tail interlobular	Tail intralobular	Head Total	Tail Total
Overall (mean +/- SD, n = 100)	20.70 ± 19.35	19.13 ± 22.49	25.77 ± 18.96	24.19 ± 23.43	19.95 ± 19.58	24.97 ± 20.25
Hispanic/Latino (mean +/- SD, n = 17)	24.56 ± 26.43	19.70 ± 24.87	32.09 ± 17.46	30.14 ± 23.82	22.13 ± 25.40	31.12 ± 19.64
White (mean +/- SD, n = 43)	20.48 ± 18.72	25.12 ± 26.05	29.23 ± 21.05	28.12 ± 25.12	22.91 ± 21.03	28.65 ± 22.00
Black (mean +/- SD, n = 32)	18.17 ± 16.77	11.04 ± 12.82	19.32 ± 16.60	18.05 ± 21.33	14.57 ± 13.24	18.68 ± 18.05
Asian (mean +/- SD, n = 6)	16.05 ± 9.64	7.26 ± 5.87	18.04 ± 8.25	10.91 ± 8.39	11.66 ± 6.79	14.53 ± 7.19
American Indian or Alaska Native (mean +/- SD, n = 1)	37.53 ± NA	36.62 ± NA	21.87 ± NA	22.36 ± NA	37.07 ± NA	22.11 ± NA
Other / Not reported (mean +/- SD, n = 1)	57.60 ± NA	56.98 ± NA	53.15 ± NA	61.51 ± NA	57.29 ± NA	57.33 ± NA

Table S2: Association of Clinically Relevant Variables With IPFD Stratified by Obesity Status

Group	Fat Compartment	HbA1c Correlation (r, p)	Diabetes Meds (mean diff, p)	Lipid Meds (mean diff, p)
**Obese (n = 51)**	Head - Interlobular	r = 0.025, p = 0.868	9.7, p = 0.328	3.8, p = 0.658
	Head - Intralobular	r = 0.067, p = 0.651	2.8, p = 0.793	5.0, p = 0.671
	Tail - Interlobular	r = –0.011, p = 0.944	7.9, p = 0.300	6.7, p = 0.600
	Tail - Intralobular	r = 0.153, p = 0.332	16.7, p = 0.131	12.4, p = 0.379
**Non-Obese (n = 49)**	Head - Interlobular	r = –0.012, p = 0.935	2.7, p = 0.675	0.2, p = 0.976
	Head - Intralobular	r = –0.079, p = 0.604	5.7, p = 0.378	1.5, p = 0.845
	Tail - Interlobular	r = 0.096, p = 0.542	–5.1, p = 0.491	0.2, p = 0.987
	Tail - Intralobular	r = –0.001, p = 0.995	–6.8, p = 0.607	–10.2, p = 0.456
